# A nucleotide-independent cyclic nitroxide label for monitoring segmental motions in nucleic acids

**DOI:** 10.1186/s13628-015-0019-5

**Published:** 2015-04-09

**Authors:** Phuong H Nguyen, Anna M Popova, Kálmán Hideg, Peter Z Qin

**Affiliations:** Department of Chemistry, University of Southern California, 840 Downey Way, Los Angeles, CA 90089-0744 USA; Institute of Organic and Medicinal Chemistry, University of Pécs, Szigetic Strasse 12, Pécs, Hungary; Current Address: Bachem Americas, Torrance, CA 90505 USA; Current Address: Department of Integrative Structural and Computational Biology, The Scripps Research Institute, La Jolla, CA 92037 USA

**Keywords:** Site-directed spin labeling, EPR, Rigid label, Nucleic acids, Dynamics

## Abstract

**Background:**

Spin labels, which are chemically stable radicals attached at specific sites of a bio-molecule, enable investigations on structure and dynamics of proteins and nucleic acids using techniques such as site-directed spin labeling and paramagnetic NMR. Among spin labels developed, the class of rigid labels have limited or no independent motions between the radical bearing moiety and the target, and afford a number of advantages in measuring distances and monitoring local dynamics within the parent bio-molecule. However, a general method for attaching a rigid label to nucleic acids in a nucleotide-independent manner has not been reported.

**Results:**

We developed an approach for installing a nearly rigid nitroxide spin label, designated as R5c, at a specific site of the nucleic acid backbone in a nucleotide-independent manner. The method uses a post-synthesis approach to covalently attach the nitroxide moiety in a cyclic fashion to phosphorothioate groups introduced at two consecutive nucleotides of the target strand. R5c-labeled nucleic acids are capable of pairing with their respective complementary strands, and the cyclic nature of R5c attachment significantly reduced independence motions of the label with respect to the parent duplex, although it may cause distortion of the local environment at the site of labeling. R5c yields enhanced sensitivity to the collective motions of the duplex, as demonstrated by its capability to reveal changes in collective motions of the substrate recognition duplex of the 120-kDa *Tetrahymena* group I ribozyme, which elude detection by a flexible label.

**Conclusions:**

The cyclic R5c nitroxide can be efficiently attached to a target nucleic acid site using a post-synthetic coupling approach conducted under mild biochemical conditions, and serves as a viable label for experimental investigation of segmental motions in nucleic acids, including large folded RNAs.

**Electronic supplementary material:**

The online version of this article (doi:10.1186/s13628-015-0019-5) contains supplementary material, which is available to authorized users.

## Background

Spin labels refer to chemically stable radicals attached at macromolecules, and are utilized to study structure and dynamics of bio-molecules in techniques such as site-directed spin labeling (SDSL) and paramagnetic NMR. In particular, SDSL monitors behaviors of spin labels using electron paramagnetic resonance (EPR) spectroscopy, and is capable of studying high-molecular-weight systems under physiological conditions using a small amount of samples. SDSL has been shown as a valuable method for investigating structure and dynamics of proteins, biological membranes, nucleic acids, and their assemblies [1-5].

Many SDSL studies use pyrroline- or piperidine-based nitroxides that are covalently attached at a specific site of the target macromolecule. These labels can be categorized into two groups based on the nature of chemical coupling between the target molecule and the nitroxide moiety bearing the unpaired electron. A large number of them fall into the “flexible” category, with the nitroxide moiety connected to the target molecule by rotatable bonds. Examples of flexible labels include the prototypic R1 label for proteins, where the pyrroline ring is connected via a disulfide bond to a cysteine [1]; and the R5 and R5a nitroxides, where the pyrroline ring is connected via single-bonds to a phosphorothioate introduced at the backbone of a nucleic acid molecule [5]. The flexible labels in general have a certain degree of adaptability, which can be advantageous in mitigating perturbations to the native conformation and in sensing structural variations at the labeling site [1]. On the other hand, they undergo independent motions with respect to the target, which may pose a challenge in correlating measurements obtained from the nitroxide with structural and dynamic features of the target molecule [3].

In contrast, rigid labels have limited or no independent motions between the nitroxide moiety and the target molecule. This can lead to a number of advantages, such as a narrow distribution in the measured inter-nitroxide distances [6-8], an enhanced ability to report orientation of aligned target molecules with respect to the external magnetic field [6,8], and a greater sensitivity to motions of the target molecule [6-8]. An example of a rigid label used in protein studies is TOAC (2,2,6,6-tetramethylpiperidine-1-oxyl-4-amino-4-carboxylic acid), which is incorporated via chemical synthesis and results in the nitroxide piperidine moiety fused directly with the peptide backbone [9]. In addition, a semi-rigid RX label has been reported, in which a pyrroline moiety is attached in a cyclic fashion to two engineered cysteine sites [6-8].

For nucleic acid SDSL, a Ç label, in which the nitroxide is rigidly fused with a modified cytosine, has been synthesized and incorporated at specific positions of either DNA [10] or RNA [11]. Ç, which completely eliminates independent motions of the nitroxide moiety with respect to the nucleobase, enhances one's capability to derive information on nucleic acid molecules from inter-Ç distance measurements [12,13] or from monitoring Ç rotational dynamics [14-16]. Nonetheless, Ç labeling involves complex chemical synthesis procedures, and is confined by the availability of cytosine in the target sequence.

**Figure 1:**
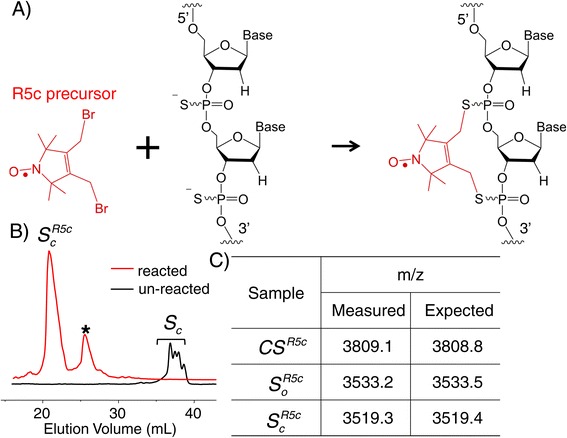
**A cyclic nitroxide label for nucleic acids. (A)** A schematic of the labeling strategy. **(B)** Characterization of R5c labeling of the *S*_*c*_ RNA by anion-exchange HPLC. The chromatographic trace for *S*_*c*_ subjected to R5c labeling is shown in red, and that for the unlabeled *S*_*c*_ is shown in black. **(C)** Characterization of R5c-labeled oligonucleotides by MALDI-TOF mass spectrometry.

Work reported here explores an alternative approach for covalently installing a nearly rigid nitroxide label at specific locations of nucleic acids in a nucleotide-independent manner. Expanding on the strategy previously developed for attaching the R5 and R5a labels to one phosphorothioate (ps) group [5,17,18], a R5c nitroxide label was efficiently attached, in a cyclic manner, to two ps groups introduced at consecutive nucleotides within a target DNA or RNA strand (Figure 1A). Upon hybridization to a complimentary strand, the cyclic nature of the R5c attachment largely eliminates independent nitroxide motions with respect to the nucleic acid duplex. The advantage of such enhanced coupling was demonstrated by the use of R5c to examine collective motions of the substrate recognition duplex in the *Tetrahymena* group I ribozyme. The results establish the applicability of R5c as a nucleotide-independent semi-rigid label for studying segmental motions in nucleic acids.

## Methods

### Oligonucleotides

Table 1 provides sequences of oligonucleotides used in this work. All synthetic oligonucleotides were obtained commercially (Integrated DNA Technology, Inc.). The L-16 ScaI variant of the *Tetrahymena* group I ribozymes, including both wild type and mutants (Additional file 1: Supporting Information, Figure S1), were produced by T7 *in vitro* transcription as previously reported [16,19].

**Table 1:** **Oligonucleotides used in this study**

**Name**	**Sequence** ^**(a)**^	**Notes**
CS	5′-dCdTdAdCdT*dG*dCdTdTdTdAdG-3′	DNA for cyclic ps attachment
CS_B	5′-dCdTdAdAdAdGdCdAdGdTdAdG-3′	Complementary to CS
*S*_*o*_^(b,c,d)^	5′-rCrCrC*mU*rC**dU**rArAdA*dC*rC-3′	RNA for cyclic ps attachment; directs ribozyme into the “open” complex
*S*_*c*_^(b,c)^	5′-rCrCrCrUrC**dU**rArAdA*dC*rC-3′	RNA for cyclic ps attachment; directs ribozyme into the “close” complex
*S*_*s*_^(b,c,d)^	5′-rCrCrC*mU*rC**dU**rArArAdC*rC-3′	RNA for single ps attachment; directs ribozyme into the “open” complex
IGS	5′-rGrGrUrUrUrGrGrArGrGrG-3′	Complimentary to *S*_*o*_, *S*_*c*_, or *S*_*s*_

(a) Definition of symbols: *: phosphorothioate modification; r: 2′-OH; d: 2′-H; m: 2′-OCH_3._

(b) 2′-H substituted at position(s) adjacent to the phosphorothioate group(s) to prevent strand scission upon nitroxide labeling [17,19,22].

(c) Bold position substituted to 2′-H to reduce ribozyme cleavage rate [19].

(d) Italicized position substituted to 2′-OCH_3_ to remove a tertiary interaction with the ribozyme core [19].

### Nitroxide labeling

R5c precursor, 3,4-bis(dibromomethyl)-2,5-dihydro-2,2,5,5-tetramethyl-1H-pyrrol-1-yloxyl radical was synthesized as reported [20]. In each 100 μL labeling reaction, 20-30 nmol of a double ps-modified crude oligonucleotide was reacted with 100 mM of the R5c precursor in 50% (v/v) acetonitrile and 100 mM MES (pH 5.8). The reaction mixture was incubated overnight under constant agitation. The reaction products were purified by anion-exchange HPLC [21]; and labeled oligonucleotides were desalted by reverse-phase HPLC. Desalted samples were lyophilized, then re-suspended in ME buffer (10 mM NaMOPS, pH 6.5, 1 mM EDTA) and stored at -20°C. The final concentration of each labeled oligonucleotide was determined by absorption at 260 nm using extinction coefficients listed in Additional file 1: Table S1.

As previously noted [17,19,22], in an RNA strand, the presence of a 2′-OH at position(s) adjacent to the phosphorothioate group(s) results in strand scission upon nitroxide labeling. In this work, this problem was overcome by 2′-H substitutions at appropriate nucleotides (see Table 1), although other substitutions (e.g., 2′-F, 2′-OCH_3_) may achieve the same goal.

### EPR sample preparation

To assemble a duplex, the R5c-labeled strand was incubated with a two-fold excess of the unlabeled complementary strand in an aqueous buffer at room temperature for 1 hour. The DNA duplex, formed by CS and CS_B strands (Table 1), was assembled in a solution containing 100 mM NaCl and 50 mM Tris-HCl (pH 7.5). RNA duplexes, formed by the IGS strand and S_c_, S_o_, or S_s_ (Table 1), were assembled in buffer A (10 mM MgCl_2_ and 50 mM NaMOPS). The annealed samples were directly used for EPR measurements.

To assemble an R5c-labeled ribozyme complex, the ribozyme (300 μL of 1 μM) was first pre-folded at 50°C for 30 min in buffer A. Appropriate amount of R5c-labeled strands dissolved in buffer A was then added to achieve a substrate/ribozyme ratio of 1:2. The final mixture was incubated at room temperature for 1 hour, then concentrated to approximately 10-20 μL using a pass-through membrane concentrator (MWCO 30 kDa, Millipore Inc.). Concentration was performed twice to ensure removal of the unbound labeled strands, and the assembled complex was immediately used for EPR measurements. The final concentration of each EPR sample ranged from 10 to 40 μM as determined by their respective absorbance at 260 nm.

### Continuous-wave EPR spectroscopy

Each EPR sample was placed in glass capillaries (0.60 mm × 0.84 mm) sealed at one end. X-band (~9.34 GHz) continuous-wave (cw-) EPR spectra were acquired at 25°C using either a Bruker EMX Spectrometer equipped with a high sensitivity cavity (ER 4118 HS, Bruker Biospin, Inc.) or an ELEXSYS E580 spectrometer equipped with an EN 4118X-MD4 resonator. The incident microwave power was 2 mW, and the field modulation was 1 to 4G at a frequency of 100 kHz. Post-acquisition data processing, including baseline correction and spectral normalization to the same number of spins, were carried out using software kindly provides by the Hubbell group of UCLA.

### Thermal denaturation measurements

Measurements were carried out using a DU800 UV-Vis spectrometer (Beckman Coulter, Fullerton, CA). Samples (1- 2 μM) were heated gradually from 6 to 80°C, and absorbance at 260 nm was continuously recorded. The measured melting curves were analyzed as described [23] to obtain the standard state enthalpy (ΔH^0^) and entropy (ΔS^0^) of the transition, from which the standard state free energy of transition at 37°C (ΔG^0^_37°C_) was computed.

### Mass spectrometry

For each mass spectrometry measurement, an oligonucleotide (30 – 40 μM) was mixed with the matrix (35 mg/mL of 3-hydroxypicolinic acid, 7 mg/mL of di-ammonium hydrogen citrate, and 15/85% acetonitrile/water) in a ratio of 1:2 (v/v). After vigorous mixing, each sample was manually deposited (in 0.5 – 1 μL droplets) onto a stainless steel sample plate (Applied Biosystems, Foster City, CA) and air-dried. MALDI-TOF measurements were carried out using a Voyager-DE STR system (Applied Biosystems), and spectra were acquired in the linear mode to monitor positives ions in the mass range of 1,500 to 10,000 Da.

## Results and discussion

### A post-synthesis scheme for incorporating a cyclic nitroxide label in nucleic acids

To incorporate the cyclic R5c, the bi-functionalized R5c precursor was reacted with a nucleic acid strand containing ps modifications introduced at two consecutive nucleotides during solid-phase chemical synthesis (Figure 1A). Figure 1B shows an example of anion-exchange HPLC traces obtained with the *S*_*c*_ RNA (Table 1). Upon R5c labeling (red trace), two major peaks eluting earlier than the unlabeled species (black trace) were observed. The species eluted first was assigned to the cyclic ${S}_c^{R5c}$, as the loss of two negative charges in the ${S}_c^{R5c}$ product would result in reduced column retention and earlier chromatographic elution. This assignment was confirmed by mass spectrometry (Figure 1C).

The second species observed in the reaction mixture (Figure 1B, marked by *) eluted later than the cyclic ${S}_c^{R5c}$ but earlier than the unlabeled strand. Based on mass spectrometry data and comparisons with the reaction product obtained using the *S*_*s*_ strand that contains a single ps (Table 1), this species was assigned as that of a R5c attached to one of the two ps groups in *S*_*c*_ in a linear fashion (Additional file 1: Figure S2). Other potential side-products, such as those corresponding to different R5c-to-RNA stoichiometry, were not observed.

Note that chemical synthesis results in two possible diastereomers (R_*p*_ and S_*p*_) at each ps modified nucleotide, and in a double-ps modified strand such as *S*_*c*_ (Table 1), there are a total of four possible stereomeric configurations, which could account for the four overlapping peaks observed in the HPLC trace (Figure 1B, black). As R5c labeling was carried out with the oligonucleotide in a highly flexible single-stranded state, and both the R_*p*_- and S_*p*_-diastereomer react with the nitroxide precursor [24], the labeled ${S}_c^{R5c}$ is likely an unresolved mixture of all four diastereomers with the nitroxide attached. Although we have previously demonstrated diastereomer separation by HPLC at certain sites in single-ps modified oligonucleotides [24], the same procedure was much less effective for the double-ps modified oligonucleotides. Other approaches (e.g., modification of chemical synthesis scheme) need to be explored should one require to separate diastereomers in the double-ps modified oligonucleotides.

In addition to *S*_*c*_, R5c labeling was successfully carried out on another RNA strand *S*_*o*_ and the DNA strand CS (Figure 1C, Table 1). Overall, the results demonstrated that our protocol efficiently produces the desired cyclic R5c-labeled oligonucleotides. We note that incorporation of the R5c label is independent of the nucleotide identity at the target site. It relies on the presence of two phosphorothioate groups, which can be installed at specific sites using a simpler synthetic scheme as compared to that required for Ç synthesis and incorporation. This enables facile production of a variety of R5c labeled nucleotides (Table 1). In principle, the two phosphorothioate groups can also been installed enzymatically, as both DNA and RNA polymerases accept [α-thio]triphosphate nucleotides. This may potentially allow R5c labeling in long nucleic acid strands that are beyond the current limit posted by chemical synthesis.

### Duplex formation with R5c-labeled oligonucleotides

**Figure 2:**
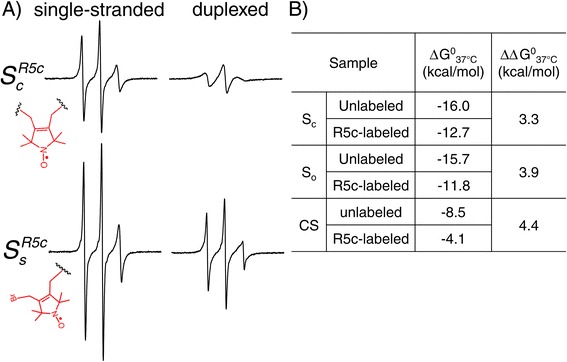
**Characterization of R5c-labeled oligonucleotide duplexes. (A)** cw-EPR spectra of ${S}_c^{R5c}$ (top, cyclic nitroxide attachment) and ${S}_s^{R5c}$ (bottom, linear nitroxide attachment). RNA duplexes were formed by pairing with the complementary IGS strand (see Table 1). **(B)** Thermal denaturation results. Errors in the reported ΔG^0^_37°C_ values were estimated to be < 1.0 kcal/mol from repeated measurements.

To examine whether cyclic attachment of R5c reduces independent motions of the nitroxide with respect to the parent molecule, we compared cw-EPR spectra of ${S}_c^{R5c}$ (i.e., *S*_*c*_ strand labeled with cyclic R5c) to ${S}_s^{R5c}$, which has R5c attached via a single ps linkage (Figure 2A). In aqueous buffer at room temperature, spectra for the single-stranded ${S}_c^{R5c}$ and ${S}_s^{R5c}$ both show three sharp lines with uneven amplitudes, although lines in the ${S}_c^{R5c}$ spectrum are broader than those for ${S}_s^{R5c}$. These spectral characteristics can be attributed to the fast global tumbling of the short oligonucleotide and a lack of structural restriction in the single-stranded state.

Addition of a complementary strand results in spectral broadening for both ${S}_c^{R5c}$ and ${S}_s^{R5c}$, reflecting reductions in global tumbling and increased structural constraints associated with duplex formation. Importantly, the ${S}_c^{R5c}$ duplex spectrum features broader lines than that of ${S}_s^{R5c}$, indicating reduced nitroxide mobility of R5c. As the underlining RNA duplexes are nearly identical, reduced motions in ${S}_c^{R5c}$ resulted from reduced rotations at bonds connecting the pyrroline moiety to the duplex. This indicates that the cyclic R5c succeeded in reducing independent motions of the nitroxide. Similar results were obtained from cw-EPR measurements of ${S}_o^{R5c}$ and *CS*^*R*5*c*^ (Additional file 1: Figure S3).

To assess R5c perturbation to the native duplex conformation, thermal denaturation measurements were carried out (Figure 2B). For the two RNAs, R5c labeling destabilized the duplex by 3.3 and 3.9 kcal/mol (Figure 2B, ${S}_o^{R5c}$ and ${S}_c^{R5c}$). These are approximately twice of the 1.6 kcal/mol value previously found for the R5a label, which was attached to *S*_*s*_ via a single ps linkage [19]. Moreover, destabilization of the *CS*^*R*5*c*^ DNA duplex was 4.4 kcal/mol (Figure 2B), which is much larger than the 0.1 – 0.6 kcal/mol values previously measured for R5a-labeled CS duplexes [25]. These data indicate that R5c labeling presents a larger degree of perturbations to duplex conformations. We note that distances between the two adjacent ps sulfurs are shorter in an A-form duplex compared to that in a B-duplex (Additional file 1: Table S2), and the A-form RNA duplexes seem to better accommodate R5c as they were destabilized to a lesser degree than the B-form DNA (Figure 2B). This suggests that in the future one may explore R5c analogs with longer distance between the reactive functional groups in order to decrease structural perturbations to nucleic acid duplexes.

In summary, R5c-labeled strands are able to form duplexes, and the cyclic nature of the label significantly reduces independent motions of the pyrroline ring, although it also presents a larger degree of perturbations to the local duplex conformation. As such, R5c is less suitable for investigating the local environment at the level of individual nucleotides, as previously demonstrated for the R5 and R5a labels [24-27]. Instead, it may be advantageous for sensing motions of “rigid body” elements, such as an RNA duplex. This is demonstrated below using a 400-nucleotide *Tetrahymena* group I ribozyme.

### Probing collective motions of the substrate recognition duplex in the Group I ribozyme

**Figure 3:**
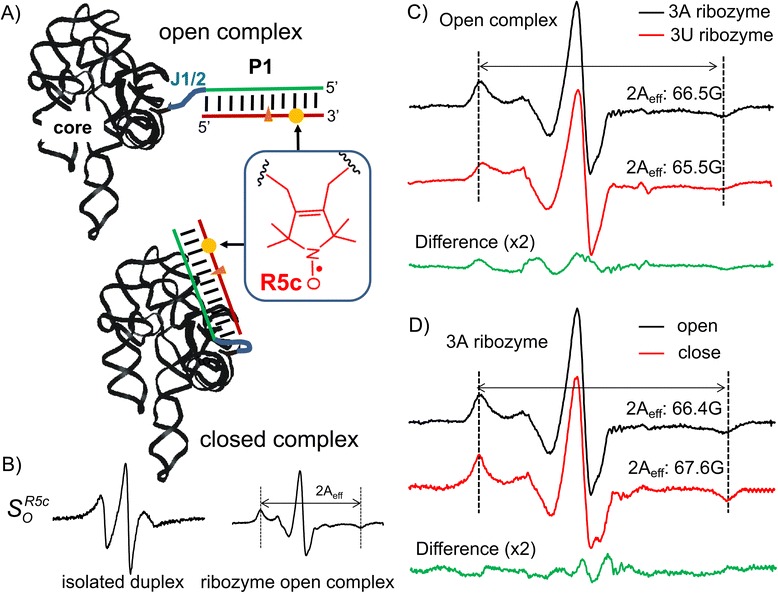
**Monitoring P1 motions in group I ribozymes. (A)** Schematic representation of the ribozyme open (top) and closed (bottom) complexes. The substrate is represented by the red line, and the cleavage site is indicated by the red triangle. R5c labeling site is marked by the yellow dot. **(B)** EPR spectra of the R5c-labeled P1 duplex in isolation and as part of the wild-type ribozyme open complex. **(C)** Spectral comparisons between wild-type (3A) and mutant (3U) ribozymes containing R5c-labeled P1. Amplitude of the difference spectra (green) was scaled by 2-fold. **(D)** Spectra of R5c-labeled P1 in the wild-type ribozyme open and closed complexes.

The *Tetrahymena* group I ribozyme is a model system for investigating structures, folding, and function of large RNAs [28]. The ribozyme recognizes its substrate by forming a duplex (designated as P1) between the substrate oligonucleotide and a single-stranded Internal Guide Sequence (IGS) within the ribozyme (Additional file 1: Figure S1). Upon P1 formation, the ribozyme first adopts a state called the open complex, in which P1 extends from the ribozyme core through a single-stranded J1/2 linker and makes no tertiary contact to the ribozyme core (Figure 3A, top). Subsequently, P1 docks into the pre-folded ribozyme core via multiple tertiary interactions, forming the “closed complex” in which the substrate is properly positioned for cleavage at the cognate site (Figure 3A, bottom). Previously, both the flexible R5a and the rigid Ç have been incorporated into P1 to monitor its dynamics [16,19]. These prior studies took advantage of the fact that the P1 segment mimicking the 5′ exon makes no direct contact to the ribozyme core, hence modifications and local perturbations at this segment do not affect studies of ribozyme structure and dynamics [19,28]. Following the same design, R5c was attached to the 5′-exon segment of P1 (Figure 3A), and its ability to report variation in P1 motions was investigated.

First, we investigated dynamics of P1 at the ribozyme open complex. The open complex with a R5c-labeled P1 was assembled by mixing ${S}_o^{R5c}$ with an excess of the wild-type ribozyme (Table 1, Figure 3B) [29]. The corresponding cw-EPR spectrum shows a broad central line and well resolved hyperfine extrema, and is drastically different from the one obtained for the isolated ${S}_o^{R5c}$ duplex (Figure 3B). This reflects reduction in nitroxide mobility arisen from reduced tumbling of the duplex as it becomes attached to the high-molecular-weight ribozyme core. Compared to SDSL data previously reported for the open complex, nitroxide mobility observed using ${S}_o^{R5c}$ is much lower than that measured using the flexible R5a [16] (Additional file 1: Figure S4), demonstrating a significant reduction in independent nitroxide motions. On the other hand, ${S}_o^{R5c}$ shows higher nitroxide mobility as compared to the completely rigid Ç (Additional file 1: Figure S4). This indicates that R5c retains a certain degree of independent motions with respect to P1, which may reflect a combination of limited bond motions in R5c and local motions in the phosphodiester backbone at or near the attachment points.

We previously showed that mutating J1/2 from “AAA” (“3A” ribozyme) to “UUU” (“3U” ribozyme) induces alterations in P1 dynamics in the ribozyme open complex, which are readily reported by the rigid Ç but not by the flexible R5a [16]. To assess sensitivity of the cyclic R5c to P1 motions, ${S}_o^{R5c}$ spectrum obtained with 3A was compared to that of 3U. At 25°C, spectral differences were observed (Figure 3C and Additional file 1: Figure S5). The ${S}_o^{R5c}$/3A spectrum showed partial splitting of the central line due to incomplete averaging of the *g*-tensors, while the ${S}_o^{R5c}$/3U spectrum showed no apparent splitting. In addition, the hyperfine splitting (2A_eff_) was slightly larger in the 3A ribozyme; and at both low- and high-field manifolds, the 3A ribozyme showed narrower peaks with higher amplitudes. Together, these spectral features indicate lower P1 mobility in the 3A ribozyme as compared to the 3U ribozyme, which is consistent with conclusions drawn from the Ç data [16]. As such, the results demonstrate that the cyclic R5c label does achieve a more rigid coupling to the P1 duplex than was previously obtained using R5a, thus enhancing our ability to monitor collective motions of P1 in the large ribozyme.

To further evaluate the capability of R5c, studies were carried out on the closed complex, in which P1 docks into the pre-folded ribozyme core via multiple tertiary interactions [28]. Using the 3A ribozyme and the R5c labeled oligonucleotide ${S}_c^{R5c}$ (Table 1), which thermodynamically favors formation of the closed complex, we obtained an EPR spectrum representing the closed complex (Figure 3D). As compared to the ${S}_o^{R5c}$/3A spectrum representing the ribozyme open complex, the ${S}_c^{R5c}$/3A spectrum shows clear indications of reduced *g*- and A-tensor averaging: a broader center line with a clear splitting; increased 2A_eff_; and narrower peaks at the low- and high-field manifolds (Figure 3D and Additional file 1: Figure S6). Collectively, these features indicate reduced R5c mobility in the closed complex, suggesting that ${S}_c^{R5c}$ -labeled P1 is able to dock into the ribozyme core, which restricts P1 motions.

Collectively, the data demonstrate that the semi-rigid R5c label is able to report on, with enhanced degree of sensitivity, nanosecond dynamics of the P1 duplex in both the open and closed complexes of *Tetrahymena* group I ribozyme.

## Conclusions

Data reported here clearly demonstrate that the cyclic R5c nitroxide can be efficiently attached to a given nucleic acid post-synthetically and under mild biochemical conditions. R5c-labeled nucleic acids are capable of pairing with their respective complementary strands, although thermal melting data indicate that the local environment at the site of labeling is likely distorted. The cyclic nature of the R5c attachment successfully reduces independent motions between the nitroxide pyrroline ring and the parent nucleic acid duplex, thus affording high sensitivity for the use of R5c to monitor collective duplex motions in RNA or DNA. This is clearly demonstrated by the finding that R5c is able to report differences in P1 motions between the 3A and 3U ribozymes, the detection of which evaded the flexible R5a label. The work established R5c as a viable label for experimental investigation of segmental motions in nucleic acids, including large folded RNAs. We also note that two-armed lanthanoid-chelating paramagnetic NMR probes linked to proteins in a cyclic fashion have been shown to enhance one’s ability to detect protein dynamics and interactions [30,31]. While work reported here focused on EPR measurements, R5c should also be applicable for paramagnetic NMR studies of nucleic acids and protein-nucleic acid complexes.

## Availability of supporting data

The data sets supporting the results of this article are included within the articles and its additional file.

## Additional file

Additional file 1:
**Supporting Information.**

## Abbreviations

SDSL: Site-directed spin labeling
EPR: Electron paramagnetic resonance
TOAC: 2,2,6,6-tetramethylpiperidine-1-oxyl-4-amino-4-carboxylic acid
Ps: Phosphorothioate
MES: 2-(N-morpholino)ethanesulfonic acid
Tris: Tris(hydroxymethyl)aminomethane
MOPS: 3-(N-morpholino)propansulfonic acid
MALDI-TOF: Matrix-assisted laser desorption/ionization – time of flight
IGS: Internal guide sequence
