# Supplementary material for: A nucleotide-independent cyclic nitroxide label for monitoring segmental motions in nucleic acids
Source: BMC Biophys. 2015 Apr 9;8:6. doi: 10.1186/s13628-015-0019-5 (PMC4404236; doi:10.1186/s13628-015-0019-5)
Supplement: Additional file 1: — Supporting Information. [file 13628_2015_19_MOESM1_ESM.pdf]

Supporting Information

A nucleotide-independent cyclic nitroxide label for monitoring segmental motions in nucleic acids

Phuong H. Nguyen[1,3], Anna M. Popova[1,4], Kálmán Hideg[2], Peter Z. Qin[1,*]

[1]Department of Chemistry, University of Southern California, Los Angeles, CA 90089-0744, USA
[2]Institute of Organic and Medicinal Chemistry, University of Pécs, Szigetic Strasse 12, Hungary
[3]Current Address: Bachem Americas, Torrance, CA 90505, USA
[4]Current Address: Department of Integrative Structural and Computational Biology, The Scripps Research Institute, La Jolla, CA, 92037, USA

*Corresponding author: LJS 251, 840 Downey Way, Los Angeles, CA 90089-0744. Tel: 1-213-821-2461; Fax: 1-213-740-0930; Email: pzq@usc.edu

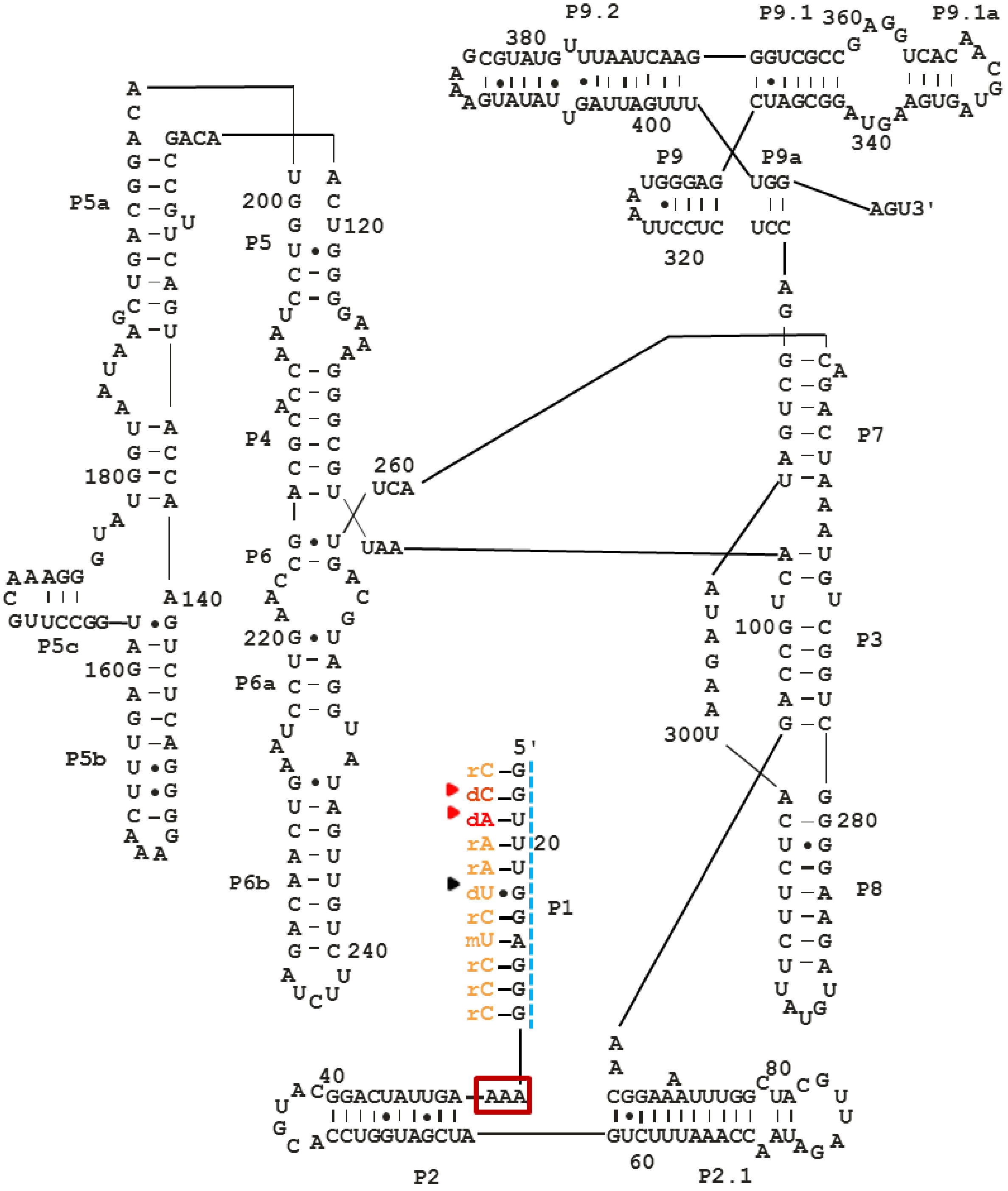

Figure S1: Secondary structure of the *Tetrahymena* group I ribozyme. An oligonucleotide substrate (in this case, S_o, in orange) base-pairs with the Internal Guide Sequence (blue dotted region) to form the P1 duplex, which is connected to the ribozyme core through the single-stranded J1/2 junction (red box). Red arrows show the phosphorothioate modification sites for subsequent R5c attachment. Black arrow shows the cleavage site. Two ribozyme variants were investigated in this work: the wild-type with a J1/2 sequence of “AAA” (also designated as “3A”); and a mutation with the J1/2 sequence mutated to “UUU” (designated as “3U”).

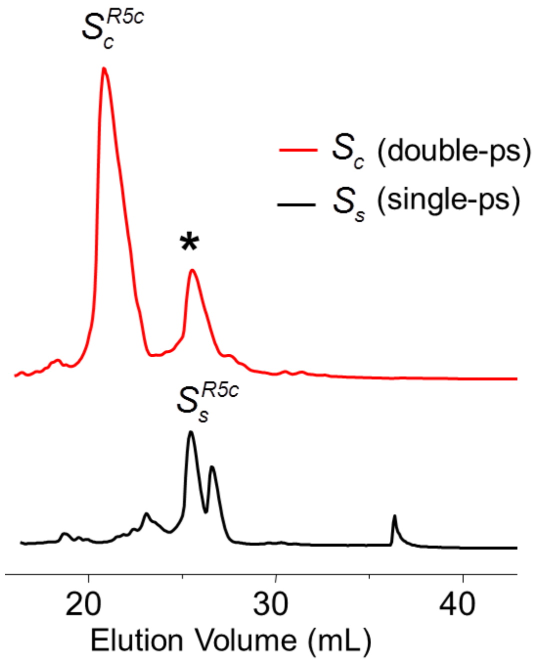

Figure S2: Characterizing side produces in the R5c labeling reactions. R5c labeling reactions were carried out with either the S_c (containing two phosphorothioates at consecutive nucleotides) or the S_s (containing only one phosphorothioate) strand. The reaction products were characterized using anion-exchange HPLC. The side-product in the S_c reaction, indicated by "*", eluted from the column at the same elution volume as the S_s product that is equivalent to R5c attached to one RNA strand through a single phosphorothioate. We note that the splitting in the S_s^{R5c} peak likely represents the resolved R_p- and S_p-diastereomers. In addition, MALDI-TOF measurement yielded a m/z value of 3520.0 for the "*" product, while a m/z value of 3600.0 is expected for a species with one R5c attached to S_c in a linear fashion. The difference of 80 units coincides with the mass of a bromide atom, and can be accounted for by the loss of the un-attached bromide functional group during the ionization process of MALDI-TOF measurement. Overall, the data support the conclusion that the "*" side-product in the S_c reaction represents one R5c connecting to the RNA in a linear fashion, although the possibility that "*" represents one of the four diastereomers of S_c^{R5c} cannot be completely excluded.

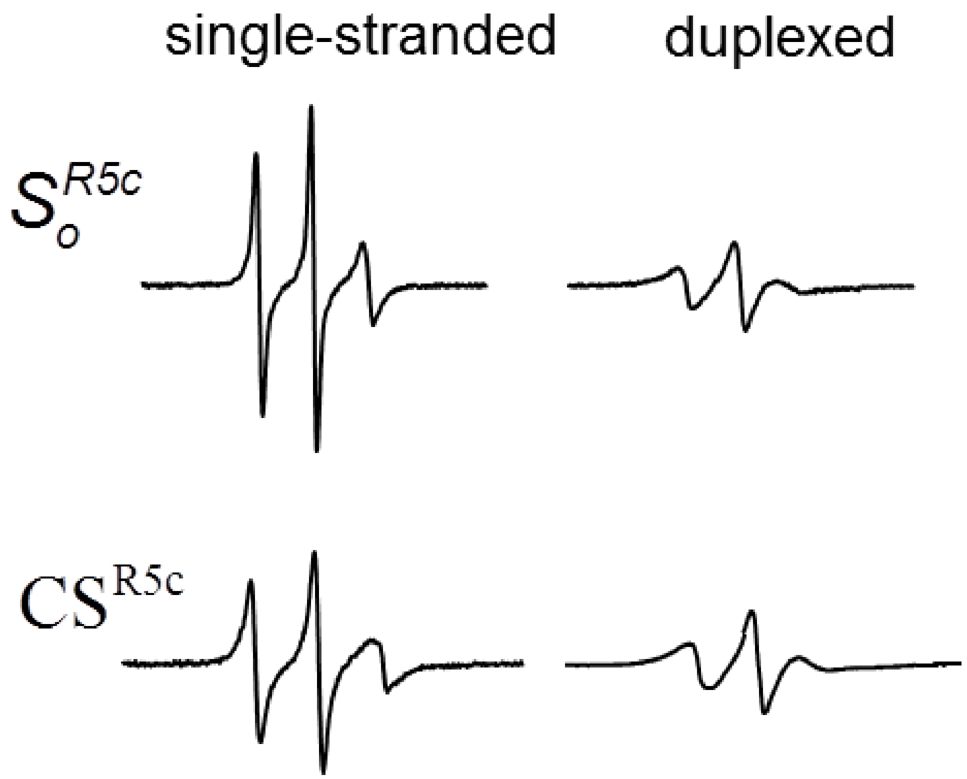

Figure S3: EPR spectra of R5c-labeled RNA (S_o, top) and DNA (CS, bottom) in single-stranded and duplexed states.

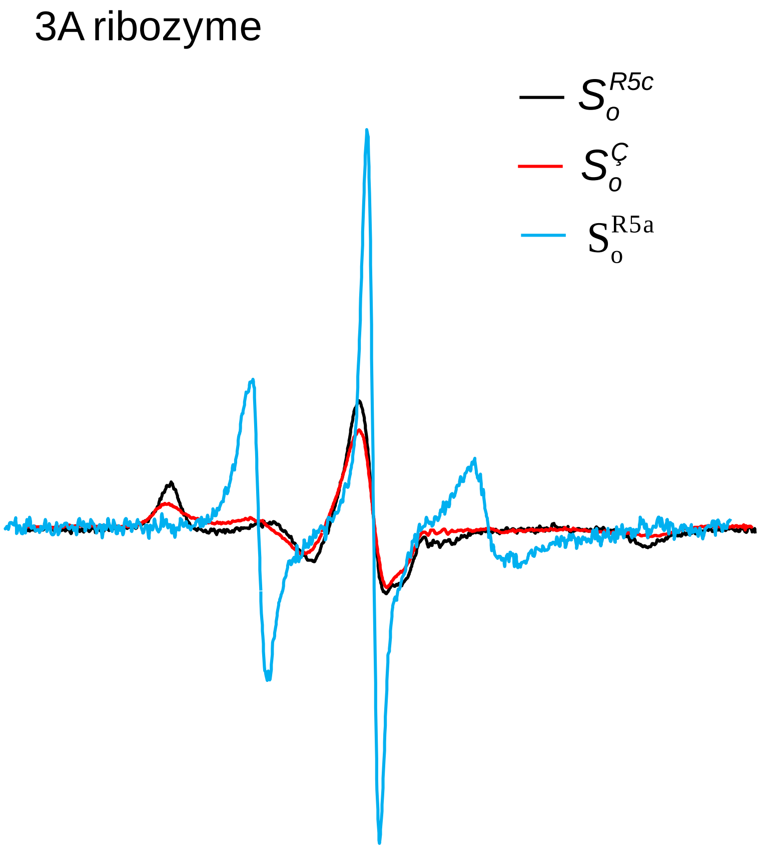

Figure S4: Comparison of spectra obtained for **Ç**-, R5c- and R5a-labeled S_o RNAs in the ribozyme open complex. The **Ç** and R5a spectra were reproduced from Nguyen *et.al.*, (2013), ChemBioChem, 14, 1720-3.

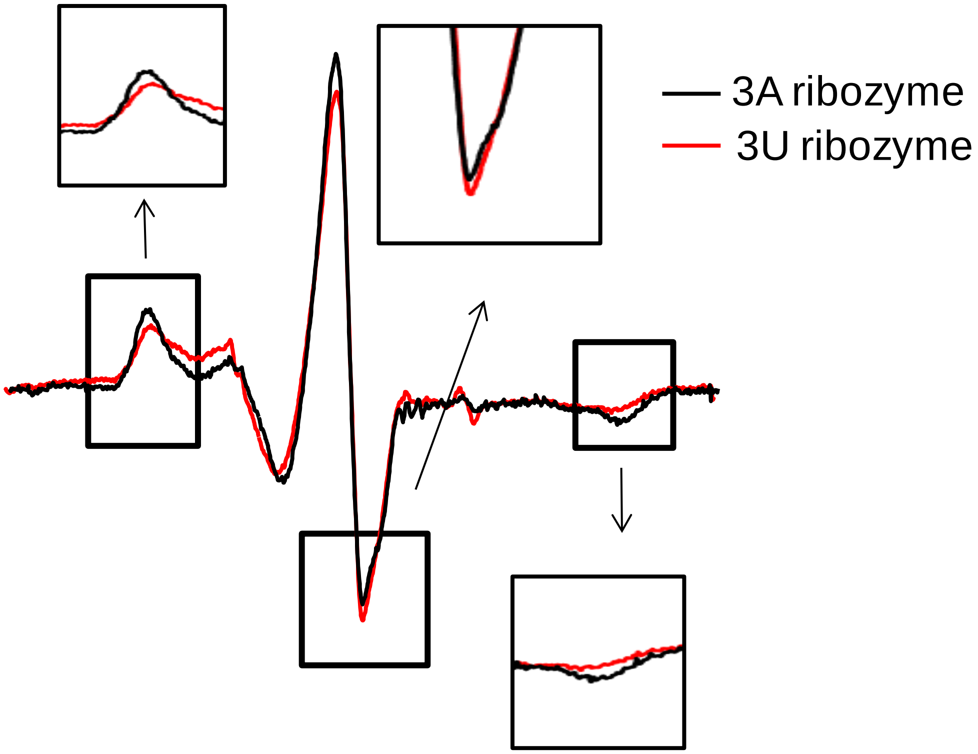

Figure S5: Overlay of R5c spectra obtained for the open complexes of the wild-type (3A) and mutant (3U) ribozyme. Insets are shown to highlight spectral differences at low-, high-, and central-field regions.

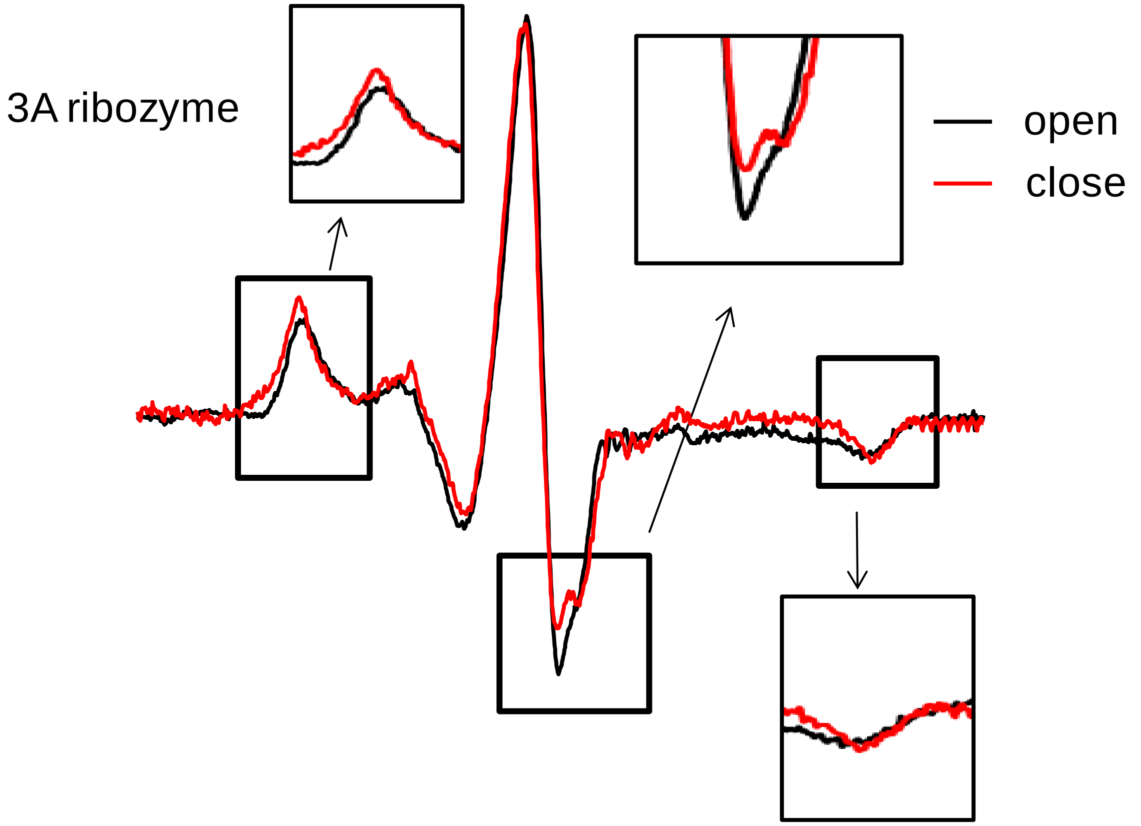

Figure S6: Overlay of R5c spectra obtained for the wild-type (3A) ribozyme open and closed complex. Insets are shown to highlight spectral differences at low-, high-, and central-field regions.

Table S1: Extinction coefficients at 260 nm (ε_{260}) for oligonucleotides used in this study.

Nomenclature[(a)]	ε_{260} ($M^{-1}\cdot cm^{-1}$)
CS	108,200
CS_B	125,800
S_o	96,700
S_c	96,700
S_s	97,900
IGS	114,400

[(a)] See sequences listed in Table 1 in the main text.

Table S2: Average distances (in Å) between non-bridging *pro*-R_p and *pro*-S_p oxygen atoms in consecutive nucleotides in nucleic acid duplexes. Data shown were obtained from 20 pairs of consecutive nucleotides in a B-DNA (1CS1.pdb, Leporc, *et.al.*, 1999, *Nucleic Acids Res.* **27:** 4759-67) and an A-RNA (1SDR.pdb, Schindelin, *et.al.*, 1995, *J. Mol. Biol*. **249:** 595-603), respectively. The analyses show distances in the A-form duplex are shorter, and may better match the span of the reactive functional groups in the R5c precursor, which is estimated to vary between 3.5 − 6.5 Å.

	pro-R_p^{n} − *pro*-R_p^{n+1}	*pro*-R_p^{n} − *pro*-S_p^{n+1}	*pro*-S_p^{n} − *pro*-R_p^{n+1}	*pro*-S_p^{n} − *pro*-S_p^{n+1}	all
B-DNA	6.8±0.2	8.4±0.2	6.7±0.2	7.6±0.2	7.4±0.7
A-RNA	5.3±0.4	7.2±0.4	5.3±0.4	6.4±0.4	6.0±0.9
